# Early Effects of Long-Term Neurotoxic Lead Exposure in Copper Works Employees

**DOI:** 10.1155/2011/832519

**Published:** 2011-05-29

**Authors:** Irina Böckelmann, Eberhard Pfister, Sabine Darius

**Affiliations:** Institute of Occupational Medicine, Otto-von-Guericke University, D-39120 Magdeburg, Germany

## Abstract

The situation of exposure in a copper works facility in Germany enabled early lead-induced neurotoxic effects to be investigated in the workers. The aim of the investigation was to study the long-term effects of small doses of lead on psychometric/psychophysiological performance of workers. The study involved 70 male lead exposed workers and 27 male controls with no neurotoxic exposure. All test persons were subjected to the method of investigation involving performance data, physiological strain data, and the subjective state. It was found that of the psychometric performance parameters, only the mainly motor performance parameters had a potential for being neurotoxic early indicators. Preferably centrally influenced performance parameters were found to be less suitable early indicators. The lead-exposed subjects exhibited a slowed poststrain resetting behaviour of the vegetative nervous system, which correlated with the individual blood lead level. This was attributed to vagus depression, which had already started in the prevailing situation of exposure and was reflected by diminished cardiac phase duration variability. Our results indicate that it is necessary to more critically choose the lead level standards in the air on the working area. Heart rate variability may be affected even at small lead concentration.

## 1. Introduction

Although the neurotoxic action of lead is known for centuries, it has not yet been adequately elucidated which effects would be useful early indicators of a clinically latent lead intoxication. Lead, as a trace element, is not necessary for the organism, and is known to be toxic in almost all organ systems. Lead, because of its high affinity for the nervous system, produces neurological effects and impairments which have been described frequently. These effects might be subdividing into those occurring in the central, the peripheral, and the autonomic nervous systems [1]. 

### 1.1. Central Nervous System (CNS)

According to reports in the literature, lead-exposed subjects have increasingly experienced CNS-induced complaints [2–15]. 

A great number of studies have focussed on deterioration of psychic or psychomental performance, describing mainly disturbed mood and affectivity [8, 16–19] as well as impaired performance such as enhanced fatigue symptoms, poor concentration, impaired memory, dysmnesia, and blocking of thought processes [9, 20]. The symptom verified most frequently has been a diminished reaction speed [9, 13, 20–22].

Neuropsychological data reported in the literature have demonstrated that continuous low-degree lead exposure impairs sensorimotor and primary central information processing. Stollery et al. [23] observed in higher-exposed workers (41–80 *μ*g/dL) a longer sensorimotor reaction time, in particular for simple tasks, and impairment of the short-term memory. Araki et al. [24] found in lead-exposed workers statistically significant changes in evoked potentials which disappeared after one year of nonexposure. Murata et al. [25], Hirata and Kosaka [26], and Abbate et al. [27] described similar effects for the early visually evoked potentials after lead exposure. For early auditory evoked potentials, latency alterations have been demonstrated after chronic lead exposure [25, 28, 29].

### 1.2. Peripheral Nervous System

In the literature, there has been evidence suggesting that the response of the peripheral nervous system to chronic lead exposure is more pronounced when compared to the CNS [26, 30–32]. Ulnaris extensor muscle paralysis in hands and feet is a typical symptom of lead intoxication.

In the search for effects of lead intoxication in the peripheral nervous system, measuring the motor nerve conduction velocity has turned out to be a useful approach even though this issue has been controversially discussed in the literature [4, 25, 26, 33–36].

As proposed by Ogawa et al. [37], determining the latency of Achilles jerk is a method that lends itself to describing lead-induced subclinical impairment of the peripheral nervous system. Investigations conducted by Stollery et al. [38] revealed delays of motor reactions in lead-exposed subjects. A marked slowing of the simple reaction time by lead was noted by Winneke [19]. Bjetak [39], in a study of lead-exposed workers, found that sensorimotor performance was affected even though memory and reaction tests did not reveal any difference compared to controls.

The effects of low-dose lead occupational exposure on neurobehavioral functions are still not well defined by occupational literature [40].

### 1.3. Autonomic Nervous System

Lead exposures may affect cardiovascular health through the autonomic nervous system [41, 42]. A method suitable to describe the function of the autonomic nervous system is the cardiac-rhythm analysis or analysis of heart rate variability (HRV) [43]. Reduced heart rate variability has been associated with sudden cardiac death and heart failure [44]. Abnormal cardiac autonomic function may be an important contributor to the pathophysiology of vascular disease, heart failure, and myocardial ischemia and their consequences, including sudden cardiac death [42].

Despite the wealth of literature published on this issue, work investigating the influence of harmful substances on this division of the nervous system has been scarce [25, 42, 45–50]. These publications gave accounts of a significantly reduced parasympathetic activity in lead-exposed workers when compared to nonexposed controls. The study from Park et al. [41] provides evidence that people with higher past exposures to lead are at increased risk of adverse health outcomes from air pollution. However, Gennart et al. [51] reported that in 98 lead-exposed workers studied, blood levels of lead (40–75 *μ*g/dL) did not exert any influence on the autonomic nervous system as judged from sinus arrhythmia. The effects of lead on the heart rate variability have not yet been established [52]. Reference [53] found that the validity and precision of the studies on the association between lead exposure and decreased heart rate variability are often limited by small sample sizes, limitations in the assessment of lead exposure, and lack of control for established cardiovascular risk factors and other confounders.

From these sources dealing with the various divisions of the nervous system and a potential effect which lead may have, in the search for early effects, it is reasonable to conclude the following.

Early forms of a neurotoxic action of lead, with no other pathological clinical findings, show numerous individual features of manifestation making the scientific description of lead-induced neuronal disorders difficult. Hence, in the search for early effects of neurotoxic lead exposure, it is only a multifactorial approach that can be pursued. The multilevel concept proposed by Fahrenberg [54], which comprehensively includes performance, strain, and subjective feeling, may serve this purpose.

Whilst in the past 30 years useful schemes of reducing the levels of harmful substances in companies and in the general environment in industrial nations have substantially reduced the lead exposure, there was a copper works facility in East Germany where workers in various jobs had been definitely continuously exposed to levels of up to 25% above the German threshold limit values (DE-MAK) of lead in air (0.1 mg/m^3^) over a period of more than ten years. The MAK value (maximale arbeitsplatz-konzentration) is defined as a maximum permissible concentration of a chemical compound present in the air within a workplace, which, according to current knowledge, does not impair the health of the employee or cause undue annoyance. Under these conditions, exposure can be repeated and of long duration over a daily period of 8 hours, constituting an average working week of 40 hours. MAK values are those from the Deutsche Forschungsgemeinschaft (DFG). For the USA and for Sweden, permissible exposure limits are 0.15 mg/m^3^ and 0.05 mg/m^3^, respectively. This rare situation of exposure, being substantially improved through rehabilitation measures after the unification of Germany, brought about the present study, as it offered a chance for objectifying neurotoxic effects induced by occupational lead exposure. 

The aim of the investigation was to define proper and sensitive indicators as screening methods of early neurologic effects after occupational exposition by lead using psychometric and psychopathologic procedures.

## 2. Subjects and Methods

The investigation schedule involved all the available male workers of a copper works facility who had had a history of several years occupational chronic lead exposure. These 70 males satisfied the criteria for being included in the study: voluntary participation, no pathological clinical findings, definite average lead exposure (0.13 ± 0.09 mg/m^3^ air) within the threshold limit value (MAK) range (see above), aged over 35 years, and not less than five years of uninterrupted work in the area of exposure. They formed the group of exposed subjects (E) with mean blood levels of lead (BPb) of 30.6 ± 10.2 *μ*g/dL over the past 12 years; the internal dose time-weighted average (TWA) calculated as proposed by Hänninen et al. [16] was 29.7 ± 10.2 *μ*g/dL. Out of the 70 exposed subjects (E), 21 had a higher exposure (hE, BPb continuously >35 *μ*g/dL) and 49 had a lower exposure (lE, mean BPb over the period under investigation <35 *μ*g/dL). The mean BPb over the 12-year period under investigation was 43.0 ± 6.1 *μ*g/dL for the hE group and 25.3 ± 6.3 *μ*g/dL for the lE group. On the day of examination, the current BPb level was 30.4 ± 15.5 *μ*g/dL in Group E, 42.9 ± 12.7 *μ*g/dL in Group hE, and 24.0 ± 12.7 *μ*g/dL in Group IE males. The TWA values for the hE and IE exposure groups were determined as 41.9 ± 6.2 *μ*g/dL and 24.5 ± 6.4 *μ*g/dL, respectively. 

On the analogy of the internal dose TWA, one can follow the procedure described by Bleecker et al. [55] to calculate the external lead-in-air lifetime-weighted average exposure (LWAE) of exposed subjects while allowing for their accurate duration of stay in the areas of exposure, along with the lead-in-air concentrations measured. The LWAE value determined for the 21 hE subjects was 0.17 ± 0.11 mg/m³ and that for the 49 lE subjects 0.10 ± 0.08 mg/m³. 

Compared to these lead-exposed workers were 27 male controls (C) working in the iron and steel industry, with no history of occupational exposure to heavy metals or solvents and with criteria for being included in the study identical to those of exposed subjects. The control group was not more similar in sample size, because we subdivided afterwards our exposed subjects into both groups, hE (*n* = 21) and lE (*n* = 49), and compared the controls with this both exposed group. 

Criteria for exclusion from the present study for both groups were evidence of nervous lesions or unusual psychic signs, known diabetes mellitus, manifest arterial hypertension or cardiac insufficiency, and abuse of alcohol and/or drugs. 

The following age information applies to the subjects studied: Group E mean age 43.4 ± 5.4 years (35–52 years), Subgroup hE mean age 41.3 ± 4.8 years (36–50 years), Subgroup lE mean age 44.3 ± 5.4 years (35–52 years), and Group C mean age 45.2 ± 4.9 years (35–52 years). Analysis of variance did not reveal any age difference between the groups.

All test persons involved were subjected to an identical test programme which included psychometry of various performance areas (1st level), determination of physiological strain reactions during the tests (2nd level), and inquiry on subjective state of health (3rd level). The test battery used the following PC systems: Swedish performance evaluation system (SPES) [56–58] and COMBITEST [59, 60].

**(1) Performance Level:**

- capacity of short-term memory (memory span for numbers and labyrinth test),
- central information processing speed (initiation time (INT) in case of single-choice reaction (SCR) to a visual signal),
- movement time (MOV) in case of single-choice reaction (SCR) to a visual signal,
- concentration power and load capacity (reaction time under selection requirements in case of a multiple-choice reaction task (MCR) with adaptive mode),
- psychomotor coordination, sensorimotor performance (outtime and speed reached in pursuit test),
- psychomotor response (maximum frequency of the preferred hand in the tapping test).

**(2) Physiological Strain Level:**

The heart action potentials were recorded by means of modified thoracic Nehb anterior leads. The R-R intervals were recorded within 1 ms accuracy. Immediately after the experiment, the process of heart period duration covering the time of the test phases identifiable by markers was visualized on monitor. Further processing of the R-R intervals by means of fast Fourier transformations (FFT) as well as calculation of the cardiac rhythm parameters and power density spectra were conducted after the test on the basis of the registered data. When plateau (steady state) was seen in cardiac performance, the following values were calculated from the R-R intervals or the cardiac phase duration (CPD) values, in addition to the heart rate (HR):

- HR as a mean heart rate value on entire recording,
- absolute sinus arrhythmia (SA_a_) as proposed by Eckoldt [61]
  (1) $${\text{SA}}_{\text{a}}=\frac{1}{n}\overset{n}{\underset{i=1}{\sum }}\left|{\text{CPD}}_{i+1}-{\text{CPD}}_{i}\right|\;\left(\text{ms}\right),$$
  where CPD = cardiac phase duration (ms) or duration of phases of cardiac cycle, *n* = number of successive CPDs considered (not less than 200), and *i* = no.  of  CPD,
- the total power density spectrum (TP) of the CPDs as a short-term estimate of the total power of spectral density in the range of frequencies between 0 and 0.5 Hz (ms^2^) representing the overall activity of the autonomic nervous system,
- the power density bands
  - VLF (0.0–0.5 Hz) = thermoregulatory band or very low-frequency band, reflecting overall activity of some slow mechanisms of sympathetic function (ms^2^),
  - LF (0.05–0.15 Hz) = circulatory band or low-frequency band, reflecting mixed sympathetic and parasympathetic activity (ms^2^),
  - HF (0.15–0.5 Hz) = RSA (respiratory sinus arrhythmia) band, or high-frequency band, reflecting parasympathetic activity and corresponds to N-N variations (time between two heartbeats) caused by respiration (ms^2^),
- the relative proportions of these bands in the total power density spectrum
  - relative UF band share of TP-VLF (%),
  - relative LF band share of TP-LF (%),
  - relative HF band share of TP-HF (%).

A major vagal influence on the SA_a_ and the HF band share in the total power density spectrum with regard to cardiac regulation has long been postulated.

Ambulatory Holter monitoring was done on a total of 70 exposed workers and 27 controls. Arrhythmia diagnosis was based on standard electrocardiographic criteria. The HRV analyses follow the guidelines published in 1996 by the HRV Task Force [62]. Ambulatory electrocardiography was obtained using a Tracker tape recorder (Reynolds Medical, Hertford, UK) at a sampling rate of 128 Hz in the occupational department.

**(3) Psychological-State Level:**

- Subjective state (EZ) as proposed by Nitsch [63, 64] and
- state during the past six months as determined by means of the SPES.

While no premorbid-intelligence determination was conducted in the subjects, there is good reason to assume that as studied by Seeber [65], it covaries with the standard of education and qualification, and the subjects studied (exposed subjects and controls) were of the same standard.In a supporting approach, a search analysis for drugs and/or metabolites including caffeine and nicotine was conducted at the University's Institute of Clinical Pharmacology, as the results of performance tests may be modified by such substances.

## 3. Statistical Analysis

All statistical analyses were performed with the statistical software package SPSS for Windows (Version 15.0). The normality of the variable distributions was evaluated. The Student's *t*-test (normal distribution) or Mann-Whitney test (not normal distribution) were applied to test statistically significant differences between workers and controls. Results were presented using tables displaying the mean with the standard deviation (SD). The analysis was performed using a critical error probability of 0.05 (5%). Moreover, statistical evaluation was performed by means of multivariate methods and single-factor analysis of variance, Mantel-Haenzel's test.

## 4. Results

Effect variables known from the literature, such as sex and circadian influence on measurements, were insignificant in this study, as it only involved males, and measurements were consistently taken at the same time of the day. 

Multivariate biostatistical analysis (ONEWAY procedure using Scheffe's test; SPSS for Windows) was performed to identify the lead exposure-related variability of the parameters studied versus other variance-generating sources (age, standard of education/qualification, prior case histories, and motivation). No statistically significant difference was found with respect to the effect factors mentioned, and hence, a monocausal consideration can be presented below excluding lead as an influencing variable.

Only essential results of the investigation are presented here.

**(1) Performance Data**

**Figure 1:**
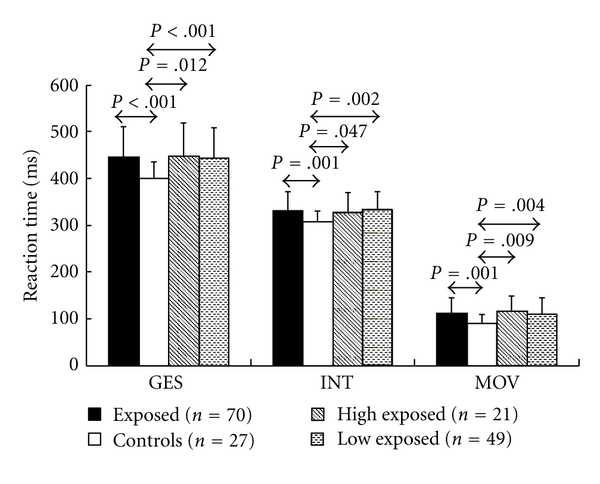
Mean values of total reaction time (GES), initiation time (INT), and movement time (MOV) of lead-exposed subjects (E) and controls (C) in the single-choice reaction task after visual stimulus.

Out of the performance data which are mainly influenced centrally, viz INT, the total duration and the number of errors in the labyrinth test, the time needed, and the interstimulus interval achieved in the adaptive MCR test (see above), it is only for the initiation time INT of the single-choice reaction task (SCR) that statistically significant differences were seen between exposed subjects and controls. The mean values of this parameter were 330.3 ± 42.8 ms for the 70 lead-exposed subjects and 306.0 ± 26.5 ms for the controls, with *P* = .001 (cf.  Figure 1), suggesting a slowing of the central information processing speed in exposed subjects.

Mainly motor performance parameters are the movement time (MOV) in the SCR, the sensorimotor performance, outtime (OUT1 and OUT2) during the first and the second halves of the pursuit test (PUR), and the maximum tapping frequency (FRQ1 and FRQ2) during the first and the second halves of the tapping test.  Table 1 shows the results of these tests. The movement time was 112.4 ± 35.4 ms in lead-exposed subjects, thus being markedly slower when compared to the controls (91.4 ± 21.6 ms) with *P* = .001. Similarly, in the tapping test, the tapping frequency of the preferred hand of exposed subjects was lower than that seen in the normal controls, in particular during the first half of the test, the respective values being 5.4 ± 0.7 Hz and 5.8 ± 0.6 Hz (*P* = .03).

The total reaction time (GES) in the single-choice reaction task comprises the preferably centrally induced INT and the motor-induced MOV. Again, exposed subjects (444.2 ± 68.1 ms) were seen to be markedly slower than the controls (398.4 ± 39.1 ms) with *P* < .001.

Selecting from the group of 70 lead-exposed subjects (E) the higher exposed (hE) and the lower exposed (lE) workers, comparison to the controls revealed significantly impaired performance for those exposed to the harmful substance as can be seen in Table 1.

**(2) Physiological Strain Data**

The subjects performing the multistage psychometric test battery did not exhibit any qualitatively different deflection of the physiological reaction parameters HR, SA_a_, and the spectral power density of the CPD as well as the arterial blood pressure. However, the controls exhibited higher average HR and lower average SA_a_ (see Table 2).

Still, care should be taken in interpreting the latter finding, as the tonicity prevailing prior to lead exposure was not known. After repeated measurements of HR and SA_a_ at rest, normal subjects can be classed into a predominantly vagotonic group I (low HR and high SA_a_) and a predominantly sympathicotonic group II (comparatively high HR and low SA_a_) [66, 67]. According to Ward's cluster analysis (SPSS), among the 70 lead-exposed copper workers, there were 32 vagotonic subjects (Group I) and 28 sympathicotonic subjects (Group II), whereas 10 subjects of Group E could not be statistically assigned. Of the 27 controls, two were classed as Group I and 22 Group II, while three could not be classed at all. Thus, a greater proportion of exposed subjects could be classed into the group of vagotonic subjects (I) which, in terms of the regulation theory, is more favourable.

**Figure 2:**
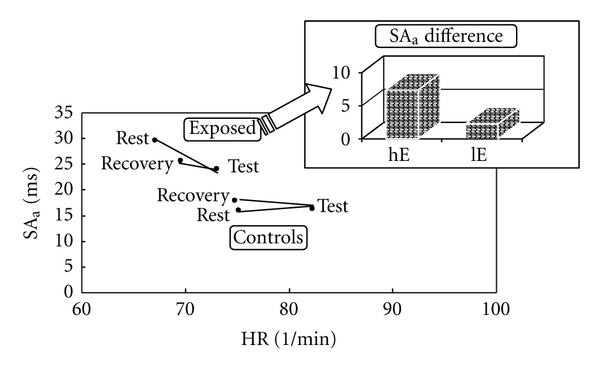
Absolute sinus arrhythmia (SA_a_) versus heart rate (HR) in lead-exposed subjects (E) and controls (C) at the three stages of rest, “memory span for numbers” test, and recovery (vegetative pattern) as well as SA_a_ differentials between rest and recovery in the two subgroups of high-exposed (hE) and low-exposed (lE) subjects.

Figure 2 shows the tonicity pattern in terms of HR and SA_a_ at rest, under the strain of the “memory span for numbers” test, and during recovery.

The mean initial condition (rest) for the two groups appears to be different: controls exhibited a high average HR of 75.2 min^−1^ and a low SA_a_ of 16.0 ms. The respective values for the lead-exposed subjects were 67.1 min^−1^ and 29.6 ms. When under strain, both groups responded with an increase in activity that was characterised by rising HR and diminishing SA_a_. Return to the initial tonicity after recovery showed differences between lead-exposed subjects and nonexposed controls. The former had a marked deficit when compared to the controls; after a 5-min recovery, they were still far away from the initial tonicity, their heart rate being 69.6 min^−1^ and their sinus arrhythmia by Eckoldt SA_a_ 25.7 ms. After an identical recovery period, the controls exhibited a much more pronounced relaxation as can be seen from the vegetatively induced cardiovascular parameters of HR = 74.8 min^−1^ and SA_a_ = 17.8 ms versus the values at rest given above. From the higher sinus arrhythmia SA_a_ during recovery versus rest, it appears that Group C was more relaxed at the end of the test series when compared to the beginning, which was not true for Group E. When Group E was subdivided into high-exposed (hE) and low-exposed (lE) subjects, it was found that the recovery tonicity, expressed as the difference between SA_a_ at rest and SA_a_ during recovery, was further away from the tonicity at rest the higher the workers' exposure to lead had been. This effect can also be seen in Figure 2.

**Figure 3:**
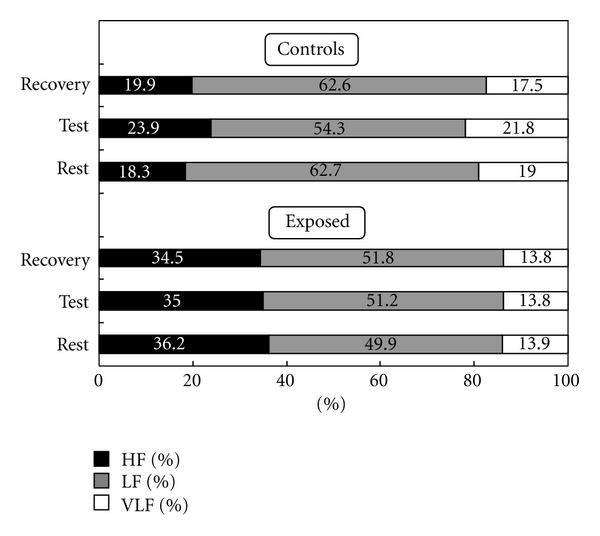
Relative frequency band percentages of cardiac phase duration variability after Fast Fourier Transform for lead-exposed subjects and controls at the stages of rest, “memory span for numbers” test, and recovery.

The result of an FFT of successive interbeat intervals represents a power density spectrum which is usually subdivided into three frequency bands A (thermoregulatory effects; 0–0.05 Hz), B (blood pressure regulation; 0.05–0.15 Hz) and C (respiratory sinus arrhythmia; 0.15–0.5 Hz). The absolute power density of the cardiac phase duration spectrum and its three bands A, B, and C, as a whole, was higher for the exposed subjects compared to the controls, a finding which is consistent with the varied group structure by the individual tonicity as outlined above. To permit a lead-modified vagal tone to be identified, one should have a closer look at the regulative dynamics of the frequency band pattern at different strain conditions. Comparison of the relative frequency bands for the two groups of subjects at rest, under test strain, and during subsequent recovery after 5 min reveals different band distributions for lead-exposed subjects and controls as demonstrated by Figure 3.

It can be seen that in the lead-exposed subjects, the relative pattern of the C band (respiratory sinus arrhythmia) did not change between the three experimental stages, whereas in the controls, a statistically significant difference (*P* = .01) was observed, typically between the test strain (23.9%) and subsequent recovery (19.9%). This was also true for the B, or cardiovascular, band. The exposed subjects did not exhibit any difference between the three test stages, but a difference did exist between rest and test (*P* = .05) as well as between test and recovery (*P* = .03).

**(3) Psychological State Data**

From the SPES questions relating to the psychological state, 16 were selected which are logically connected with a neurotoxic exposure in question: (1) = “physically tired in the morning,” (2) = “mentally tired in the morning,” (3) = “general sensation of lack of energy,” (4) = “feelings of vertigo or fainting,” (5) = “lack of initiative,” (6) = “difficulties falling asleep,” (7) = “disturbed sleep,” (8) = “waking up too early,” (9) = “finding it hard to concentrate,” (10) = “anxious, restless, out of balance,” (11) = “being forgetful,” (12) = “feeling down without reason,” (13) = “being easily upset,” (14) = “headaches,” (15) = “feeling clumsy or shaky,” and (16) = “prickling sensation, numbness in limbs.” The answers of “never”, “occasionally”, “often”, and “very often” scored points from 0 to 3. A statistically significant difference between exposed subjects and controls was noted for questions 1, 2, 3, 4, 9, 11, 12, and 15 (*P* < .05; see Table 3). Each of these significant differences concerned greater complaints experienced by the lead-exposed subjects versus controls.

At the end of the psychometric test series, each subject was shown Nitsch's EZ self-rating scale. In evaluating the subjective opinions, a positive exertion attitude too was considered as this feature is known to have a major influence on psychometric test results. Yet, similar to the “motivation” factor, no statistical differences were observed between the groups and, hence, the varied findings obtained in the performance tests are not thought to be attributable to differences in exertion attitude or motivation.

## 5. Discussion

In the light of the great expectations the occupational medicine is to meet when it comes to detecting vocationally induced disturbances of health in good time such that primarily successful preventive action can be taken, early indicators hitherto not considered from the clinical viewpoint are also needed for neurotoxic substances. This approach is in line with workers' increasing demand for comprehensive occupational medical care. 

In East Germany, an old nonferrous metal works facility was available for the investigation in which, “ideally suited” for the study, 70 male workers had been continuously exposed to occupational lead over not less than 5 years, with exposure levels being roughly identical to the current German threshold limit value for lead of 0.1 mg/m³. Of these 70 males, 21 had had a verified permanent lead concentration of more than 35 *μ*g/dL over the past 5–10 years. The average level among the normal population of Germany has been reported to range from 5 to 8 *μ*g/dL [68], and there has been a trend towards lower concentrations. 

Considering the literature on psychometric performance impairment, the lead-exposed subjects examined in the present study were expected to exhibit effects which relate to the short-term memory, the discrimination capacity, or the speed of information processing as well as the motor reaction time. In fact, only few of the present performance findings came up to the hypothetical expectations of being early indicators of a lead-induced neurotoxic harm. Those were predominantly the motor performance features of movement time in the single-choice reaction task and the tapping frequency in the tapping test, the total reaction time in the single-choice reaction task, and the initiation time, which may be considered a parameter of central information processing. The present findings failed to meet the great expectations for the adaptive multiple-choice reaction task. This contrasts sharply with Lilienthal et al. [69] who described multiple-choice reaction tests as providing more meaningful information in case of a lead-induced damage in question compared to single reactions. However, the present psychometric findings revealing an impaired performance for some of the lead-exposed subjects compared to the controls need to be qualified when the doses involved are considered. Indeed, an increasing individual lead dose did not bring about a statistically significant impairment of performance data. It is because of the pronounced difference between groups that early diagnosis in occupational medicine cannot do without single-reaction and tapping tests.

To objectify a lead-induced neurotoxic damage not yet identified by the clinician or occupational physician, use should be made of physiological strain parameters as well as psychological state and subjective experience data, in addition to the performance data referred to above.

According to the hypothesis proposed, workers after many years of lead exposure were assumed to exhibit greater strain reactions than the nonexposed controls, provided the performance was comparable. While the specificity of the strain parameters of heart rate was partly to characterise a varied strain pattern, results were obtained for the cardiac rhythm response (heart rate variability) which were not expected when the study was planned on the grounds of the literature. This relates to the slowed restoration of the initial vegetative condition in the lead-exposed subjects once they have performed their test tasks. In fact, a simple vegetative tonicity comparison between exposed subjects and controls at rest does not consistently lend itself to indicating the expected effect of a restricted cardiac phase duration variability and, thus, vagus depression by lead. In this respect, we go against a number of other workers [25, 45–47] who described this effect for lead workers at rest. In the latter publications, it has been tacitly assumed that the restricted HRV found was solely caused by exposure to heavy metals, while disregarding the fact that even normal subjects not occupationally exposed to harmful substances are generally known to differ greatly from each other because of their HRV at rest, whether inherited and/or acquired as a result of their conduct of life (in particular sports activities). Therefore, unless sufficient previous knowledge is available, sectional comparison of HRV variables for various groups of subjects is not suited to provide meaningful information on the effect of an individual factor. The effect of a slowed restoration of the initial vegetative condition as noted in this study does not take into account the interindividually varied vegetative tonicity at rest, and hence, it is believed to be a measure more appropriate to describe lead-induced vagus depression. A slower adjustment of cardiovascular parameters due to diminished vagus efferences was described as early as 1977 by Schwarz [70] who performed vagus blocking experiments.

Andrzejak et al. [71] described that in copper smelters, occupational exposure to lead HRV is lower than in healthy subjects, which results rather from the decreased parasympathetic than from the increased sympathetic activity. 

A longitudinal study performed at intervals of several years in case of persisting exposure would offer another approach to demonstrating the effect of vocationally absorbed lead. In fact, a repeated study conducted among workers of a company after about 4 years revealed a progressive restriction of the HRV [50].

As already mentioned, a study also included search for early indicators of a clinically concealed lead-induced neurotoxic damage at the level of subjective state and experience too. The hypothesis of an impaired subjective sensation of the state of health and the mental state was confirmed for a number of categories in lead-exposed workers versus controls, and the findings were statistically significant. In this context, effects with regard to tiredness (or lassitude) and lack of energy deserve particular mention. This is essential to valuation of limits as the copper workers studied had been exposed to the German threshold limit value (DE-MAK). However, verification of impaired subjective state and experience through years of exposure to lead within statutory limits does not apply to all of the categories included in the questionnaire or factor groups of the SPES method as well as Nitsch's self-rating scale. Still, applied occupational medicine should include these methods in its activities of monitoring lead-exposed subjects, especially since they can be implemented without any need for major equipment. It is admitted, though, that studies of subjective state and mental experience alone are not sufficient to reliably ascertain the neurotoxic effect of lead within the bounds of exposure hitherto considered harmless. 

Some studies described the bone lead levels as a good indicator of exposure and lead toxic effects [41] but unfortunately not possibly practicable in the occupational studies in our institute. Park et al. [44] have reported that associations between patella bone lead levels on heart rate variability are stronger among study participants with metabolic syndrome and with individual component of metabolic syndrome. 

As the occupational and environmental medicine is increasingly required to advance into spheres where marked effects by harmful substances fail to occur (being undisputable that in companies and in the general environment, weak pollutant-induced effects occur, particularly in sensitive subjects), an interdisciplinary approach involving several clinical and theoretical disciplines is becoming essential. In the present study, an exemplary attempt was made to get psychophysiology, clinical psychology, and cardiology involved in an issue of occupational medicine. Modern occupational medicine should proceed along these lines so as to meet the self-set high standard of optimal prevention for all working people.

## Figures and Tables

**Table 1:** Statistically significant performance difference between lead-exposed subjects (E) involving the subgroups of high exposed (hE) and low exposed (lE), and controls (C) in the tapping test and in the single-choice reaction task.

Test performance	Exposed (E)	Controls (C)	High exposed (hE)	Low exposed (lE)	*P* (E-C)	*P* (hE-C)	*P* (lE-C)
*Tapping*							
FRQ1 (frequency 1. Half of test) (Hz)	5.42 ± 0.69	5.78 ± 0.64	5.42 ± 0.56	5.42 ± 0.74	.030	.056	.049

*Single form visual reaction test*							
GES (total time) (ms)	444.2 ± 68.1	398.4 ± 39.1	446.0 ± 73.8	443.5 ± 66.4	<.001	.012	<.001
INT (initiation time) (ms)	330.3 ± 42.8	306.0 ± 26.5	327.1 ± 44.9	331.6 ± 42.3	.001	.047	.002
MOV (movement time) (ms)	112.4 ± 35.4	91.4 ± 21.6	115.7 ± 35.4	111.0 ± 35.6	.001	.009	.004

**Table 2:** Comparison of a number of cardiovascular parameters of lead-exposed subjects (E) involving the subgroups of high-exposed (hE) and low-exposed (lE), and controls (C) at rest and during recovery.

Physiological strain data	Exposed (E)	Controls (C)	High exposed (hE)	Low exposed (lE)	*P* (E-C)	*P* (hE-C)	*P* (lE-C)
HR rest (1/min)	67.1 ± 10.9	75.2 ± 11.7	64.4 ± 10.9	68.3 ± 10.7	.002	.003	.015
SA_a_ rest (ms)	29.6 ± 17.6	16.0 ± 16.6	34.1 ± 21.2	27.5 ± 15.5	.001	.002	.005
LF (%) rest	49.9 ± 15.8	62.7 ± 16.1	48.7 ± 14.6	50.5 ± 16.4	.001	.005	.004
HF (%) rest	36.2 ± 17.4	18.3 ± 15.3	35.8 ± 16.4	36.4 ± 18.0	<.001	.001	<.001
HR recovery (1/min)	69.6 ± 9.1	74.8 ± 13.1	67.6 ± 10.4	70.5 ± 8.5	.068	.045	.137
SA_a_ recovery (ms)	25.7 ± 12.9	17.8 ± 13.9	26.7 ± 14.6	25.3 ± 12.2	.010	.037	.019
HF Recovery (ms^2^)	4067.5 ± 3545.7	1627.5 ± 2789.7	3885.5 ± 3673.1	4152.4 ± 3523.7	.001	.019	.001
HF (%) Recovery	34.5 ± 23.9	19.9 ± 17.7	30.5 ± 23.2	36.3 ± 24.3	.002	.079	.002

**Table 3:** Results of the survey (using the SPES method) of complaints experienced by lead-exposed subjects (E) involving the subgroups of highexposed (hE) and lowexposed (lE), and controls (C) during the past six months. In the 16 answers: 1 denoted “occasional”, 2 “often”, and 3 “very often ” (The answers of 1, 2, and 3 to the particular question were percentages within a group; the balance of 100% did not complain of such symptoms).

Parameter		Exposed (E)	Controls (C)	High exposed (hE)	Low exposed (lE)	*P* (E-C)	*P* (hE-C)	*P* (lE-C)
SPES1 (physically tired in the morning)	1	51.4	22.2	42.9	55.1	<.001	.025	<.001
	2	10.0	3.7	9.5	10.2			
	3	5.7	0	4.8	6.1			
SPES2 (mentally tired in the morning)	1	2.29	0	19.0	24.5	.005	.019	.004
	2	14.3	0	0	2.0			
	3	0	0	0	0			
SPES3 (general sensation of lack of energy)	1	44.3	18.5	42.9	44.9	.006	.029	.008
	2	2.9	0	4.8	2.0			
	3	14.3	0	0	2.0			
SPES4 (feelings of vertigo or fainting)	1	41.4	14.8	47.7	38.8	.017	.057	.023
	2	5.7	3.7	4.8	6.1			
	3	4.3	3.7	0	6.1			
SPES5 (lack of initiative)	1	42.9	25.9	42.9	42.9	n.s.	n.s.	n.s.
	2	2.9	3.7	4.8	2.0			
	3	0	0	0	0			
SPES6 (difficulties falling asleep)	1	24.3	14.8	9.5	30.6	n.s.	n.s.	n.s.
	2	8.6	18.5	14.3	6.1			
	3	10.0	0	14.3	8.2			
SPES7 (disturbed sleep)	1	32.9	14.8	14.3	40.8	n.s.	n.s.	n.s.
	2	11.4	14.8	28.6	4.1			
	3	5.7	0	9.5	4.1			
SPES8 (waking up too early)	1	22.9	22.2	23.8	22.4	n.s.	n.s.	n.s.
	2	15.7	3.7	9.5	18.4			
	3	8.6	7.4	9.5	8.2			
SPES9 (finding it hard to concentrate)	1	57.1	10.2	57.1	57.1	.003	.011	.006
	2	5.7	3.7	9.5	4.1			
	3	14.3	3.7	0	2.0			
SPES10 (anxious, restless, and out of balance)	1	24.3	11.1	28.6	22.4	n.s.	n.s.	n.s.
	2	4.3	3.7	0	6.1			
	3	0	0	0	0			
SPES11 (being forgetful)	1	60	16.3	61.9	59.2	<.001	.003	.002
	2	10	3.7	14.3	8.2			
	3	2.9	0	0	4.1			
SPES12 (feeling down without reason)	1	25.7	0	38.1	20.4	.011	.004	.028
	2	2.9	3.7	0	4.1			
	3	0	0	0	0			
SPES13 (being easily upset)	1	38.6	25.9	38.1	38.8	n.s.	.059	n.s.
	2	7.1	3.7	9.5	6.1			
	3	4.3	0	9.5	2.0			
SPES14 (headaches)	1	30.0	14.8	33.3	28.6	.094	.078	n.s.
	2	5.7	3.7	9.5	4.1			
	3	5.7	3.7	4.8	6.1			
SPES15 (feeling clumsy or shaky)	1	22.9	3.7	23.8	22.4	.034	n.s.	.027
	2	4.3	0	0	6.1			
	3	14.3	3.7	0	2.0			
SPES16 (prickling sensat., numbness in limbs)	1	41.4	40.7	47.7	38.8	n.s.	n.s.	n.s.
	2	2.9	7.4	0	4.1			
	3	7.1	3.7	9.5	6.1			
